# Engineering SrTiO_3_ Nanostructures for Enhanced Photocatalytic Performance: Unveiling the Influence of Titanium Precursors and Synthesis Temperature

**DOI:** 10.1021/acsomega.5c04899

**Published:** 2025-08-28

**Authors:** Anderson Thesing, Lara F. Loguercio, Edjan Alves da Silva, Gabriel Franciosi, Arturo B. L. Véliz, Muhammad R. K. Khattak, Alexandre G. Brolo, Marcos J. L. Santos, Jacqueline F. L. Santos

**Affiliations:** † Institute of Physics, Universidade Federal do Rio Grande do Sul, Av. Bento Gonçalves 9500, Bairro Agronomia, Porto Alegre, RS 91501-970, Brazil; ‡ Institute of Chemistry, Universidade Federal do Rio Grande do Sul, Av. Bento Gonçalves 9500, Bairro Agronomia, Porto Alegre, RS 91501-970, Brazil; § Department of Chemistry and Center for Advanced Materials and Related Technologies, 8205 University of Victoria, P.O. Box 3065, Victoria, British Columbia V8W 3 V6, Canada

## Abstract

The development of advanced functional materials relies on key properties such as morphology, crystallinity, and electronic structure. In this work, we present the hydrothermal synthesis of SrTiO_3_ nanoparticles using amorphous titanium as a precursor and systematically investigate the influence of synthesis temperature (from 20 to 200 °C) on their structural, morphological, and chemical characteristics. Electron microscopy revealed a temperature-driven morphological transition from nanocube-like to spherical-like structures. X-ray diffraction analyses demonstrated improved crystallinity with increasing temperature, although local imperfections persisted, contributing to structural disorder. UV–vis spectroscopy showed a slight variation in the optical band gap, ranging from 3.36 to 3.28 eV across the samples. Notably, the sample synthesized at 60 °C exhibited significantly enhanced photocatalytic activity for H_2_ production, reaching approximately 43 μmol h^–1^. This enhancement was attributed to a synergistic interplay among the surface area, crystallinity, and composition. A dissolution–precipitation mechanism is proposed to explain the *in situ* formation of SrTiO_3_, guided by the solubility and surface reactivity of the titanium precursor. These findings provide valuable insights into the design and optimization of SrTiO_3_-based materials for photocatalytic and related applications, where fine-tuning structural and surface properties is essential to maximize performance.

## Graphical abstract

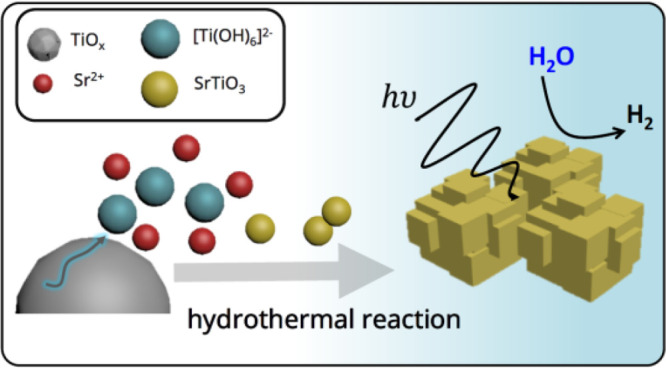

## Introduction

Perovskite-type oxides have emerged as highly versatile functional materials, exhibiting tunable structural, electronic, and catalytic properties that enable a wide range of applications, including photocatalysis, electrocatalysis, and energy storage.^1–5^ Among them, strontium titanate (SrTiO_3_) stands out due to its remarkable stability, chemical tunability, and suitable band structure for hydrogen evolution reactions under light irradiation.^4,5^ Its band structure enables UV light absorption and facilitates photoinduced charge separation, making it well-suited for heterogeneous catalytic processes, such as solar-driven water splitting and environmental remediation.^4,6–10^ However, precise control over crystallinity and morphology is essential to fully exploit these properties in practical applications. A comprehensive understanding of how these structural factors influence the material’s optoelectronic behavior is then necessary.

Conventional synthesis methods for SrTiO_3_, including solid-state reactions,^11^ sol–gel processes,^12^ and coprecipitation,^3^ typically require high temperatures and extended reaction times, often yielding large, agglomerated particles with limited control over crystal structure and defect formation. In contrast, hydrothermal synthesis offers a versatile and energy-efficient alternative, enabling the production of well-defined SrTiO_3_ nanostructures with tunable particle size, morphology, and electronic properties.^13–15^

A critical factor in SrTiO_3_ synthesis is the choice of titanium precursor. Different precursors, such as titanium butoxide, titanium chloride, titanium oxysulfate, and various polymorphs of titanium oxide (amorphous, anatase, rutile), possess distinct structural and chemical properties that significantly influence reactivity and transformation pathways during hydrothermal treatment.^15,16^ For instance, Thesing et al.^17^ reported the solvothermal synthesis of hierarchical flower-like nanostructures using titanium butoxide at 160 °C for 3 h, followed by postsynthesis thermal treatment (300–1000 °C) to achieve phase transformation into SrTiO_3_. They demonstrated that poly­(vinylpyrrolidone) (PVP) molecules acted as structure-directing agents by preferentially adsorbing on {110} facets, thereby promoting the growth of {100} facets and forming hierarchical architecture. Morphological evolution occurred at elevated temperatures due to the thermal decomposition of PVP.

Similarly, Silva et al.^18^ studied the influence of titanium chloride and titanium oxysulfate on SrTiO_3_ synthesis under microwave-assisted hydrothermal conditions at 140 °C for 10 min. Their findings revealed that titanium chloride facilitated the formation of spherical nanoparticles with reduced defect density, leading to reduced charge carrier recombination rates.

Understanding these precursor-dependent mechanisms is essential for optimizing synthetic protocols to tailor material properties. Synthesis temperature strongly influences the crystallinity, morphology, and defect structure of SrTiO_3_, thereby modulating its electronic and catalytic performance.^14–17,14–17,19–21^

In this study, we present a comprehensive investigation of the hydrothermal synthesis of SrTiO_3_ nanoparticles using different titanium oxide polymorphs (amorphous, anatase, and rutile) as precursors. Our primarily objective is to elucidate the influence of synthesis temperature on the structural, optical, and photocatalytic properties of the resulting materials for H_2_ production. The synthesis was carried out at temperatures ranging from 20 to 200 °C, and the products were thoroughly characterized.

Unlike conventional approaches that typically require high temperatures or postsynthesis treatments to achieve crystallinity and catalytic activity, our method enables the direct formation of active SrTiO_3_ nanostructures at temperatures as low as 60 °C. This low-temperature route offers an energy-efficient, scalable, and environmentally favorable alternative for the design of photocatalyst. Notably, the sample synthesized at 60 °C exhibited the highest hydrogen evolution rate, attributed to a synergistic interplay between reduced particle size, increased surface area, and a moderate degree of structural disorder. Therefore, these findings highlight that controlled disorder, preserved at low synthesis temperatures, can enhance interfacial charge transfer and overall photocatalytic performance. Additionally, the use of amorphous TiO_2_ (am-TiO_*x*_) as a precursor provides a new route for tailoring the nucleation and growth mechanisms via homogeneous nucleation pathways.

## Materials and Methods

### Materials

Titanium­(IV) butoxide (Ti­(OBu)_4_, 97%), strontium hydroxide octahydrate (Sr­(OH)_2_.8H_2_O, 95%), and sodium hydroxide (NaOH, 98%) were purchased from Sigma-Aldrich. Ultrapure water (Milli-Q) was used for all experiments.

### Methods

#### Synthesis of Amorphous, Anatase, and Rutile TiO_2_

Amorphous TiO_*x*_ (am-TiO_*x*_) was synthesized by adding 15 mL of Ti­(OBu)_4_ to 150 mL of ultrapure water under constant stirring for 2 h. The resulting mixture was centrifuged three times at 2,500 rpm for 5 min and washed with ultrapure water, followed by drying in an oven at 50 °C for 12 h. To obtain anatase TiO_2_ (A-TiO_2_) and rutile TiO_2_ (R–TiO_2_), the am-TiO_*x*_ sample was thermal treated at 420 or 700 °C, respectively, for 2 h, using a heating rate of 4 °C min^–1^. The structural characterization for A-TiO_2_ and R-TiO_2_ are provided in the Supporting Information (Figure S1).

#### Synthesis of SrTiO_3_ from Polymorphic TiO_2_

The synthesis of SrTiO_3_ was carried out in a 170 mL custom-made Teflon-lined reactor with temperatures ranging from 20 to 200 °C with a controlled heating rate of 4 °C min^–1^. The Sr­(OH)_2_·8H_2_O and Ti precursor solutions were combined maintaining a fixed volume of 60 mL and a TiO_2_ molar concentration of 5.00 mmol L^–1^. When required, NaOH was added to adjust the medium to a final concentration of 0.01 mol L^–1^. Table S1 summarizes experimental conditions used for the synthesis of SrTiO_3_ from polymorphic TiO_2_. The solution was stirred continuously for 30 min prior to the hydrothermal reaction of 12 h. All samples were centrifuged five times at 2,500 rpm for 5 min and washed with ultrapure water, followed by drying in an oven at 50 °C for 12 h.

### Characterization

XRD measurements were performed on a Siemens D500 diffractometer over a range of 10° to 80°, with a step size of 0.05° min^–1^. Analyses were carried out using CuKα radiation (λ = 1.54 Å). The lattice parameters were obtained by correlating Bragg’s law (eq 1) with the equation relating the interplanar distance to the lattice parameter a_0_ for the hkl planes in a cubic system (eq 2).

$$d_{hkl}=\frac{n\lambda }{2\sin\theta }$$ 1

$$d_{hkl}=\frac{a_{0}}{\sqrt{\left(h^{2}+k^{2}+l^{2}\right)}}$$ 2

The Scherrer eq (eq 3) was used to calculate the average crystallite size in the hkl direction (D_hkl_):

$$D_{hkl}=\frac{K\lambda }{\beta \cos\theta }$$ 3

where K is the Scherrer constant (0.89), and β is the full width at half-maximum (fwhm) of the peak corresponding to the hkl plane.

Transmission Electron Microscopy (TEM) analyses were performed using a JEOL JEM 1200 ExII microscope, operating at 80 kV. High-resolution TEM (HRTEM) analyses were conducted with a JEOL JEM 2010 at 200 kV. Scanning Electron Microscopy (SEM) analyses were performed using a Zeiss Auriga microscope operating at 2 kV. Diffuse Reflectance Spectroscopy (DRS) was carried out in a Cary 5000 spectrometer equipped with an integrating sphere, covering from 200 to 800 nm range. The optical band gap (E_gap_) was estimate using the Kubelka–Munk plot (eq 4):

$$F\left(R\right)=\frac{{\left(1-R\right)}^{2}}{2R}$$ 4

where R is the reflectance.

Raman Spectroscopy was performed using a Renishaw Raman confocal microscope equipped with a 633 nm He–Ne laser (Melles Griot) and a 20x objective lens (Leyca Microsystems). The X-ray Photoelectron Spectroscopy (XPS) was performed using an Omicron Gmbh system, using Al Kα (1486.6 eV) as the excitation source, with an analyzer pass energy of 50 eV and a step size of 1.0 eV. For the high-resolution spectra, the pass energy was 10 eV and a step size of 0.1 eV.

### Photocatalytic Activity Evaluation

Prior to the photocatalytic experiments, RuO_*x*_ cocatalyst was deposited onto SrTiO_3_-based samples using a custom-built magnetron sputtering system. A 0.2 g portion of the sample was placed in a tailor-made vibrating sample holder, which operated based on an electromagnetic oscillator at a frequency of 32.0 Hz and a current of 1.2 A. The deposition was carried out using a high-purity Ru target (99.99%) and argon (99.999%) as the sputtering gas, under a working pressure of 2.0 × 10^–3^ mbar and a power of 55 W for 2.25 min. The photocatalytic hydrogen (H_2_) evolution was performed in a gastight, side-irradiated quartz reactor. For each reaction, 50.0 mg of photocatalyst was dispersed in 50.0 mL of a 10% (v/v) aqueous methanol solution under continuous stirring. Prior to irradiation, the system was purged with high-purity argon (99.999%) to remove dissolved oxygen. The suspension was then irradiated using a Xenon (Xe) lamp equipped with a quartz filter and an AM 1.5G filter. The temperature was kept at 25 °C with a thermostatic bath. The power density was 300.0 mW cm^–2^. Hydrogen production was quantified using a Shimadzu GC-2014 gas chromatograph, with argon as the carrier gas.

## Results and Discussion

The SrTiO_3_ was synthesized using either amorphous or crystalline TiO_2_ (anatase or rutile), and the XRD results are presented in Figure S2a. By analyzing the ratio of the precursor to the main SrTiO_3_ peak intensities (2θ ∼ 25.3° for A-TiO_2_, 2θ ∼ 27.5° for R-TiO_2_, and 2θ ∼ 32.2° for am-TiO_*x*_), improvements in the reaction yield for the conversion to SrTiO_3_ are observed when using am-TiO_*x*_ as the precursor.

To elucidate the formation mechanism and predict the properties and behavior of the nanoparticles for desired applications, it is essential to understand the underlying synthesis process. Eckert Jr. et al.^22^ propose two possible pathways for the BaTiO_3_ synthesis mechanism, which are described as (i) *in situ* and (ii) dissolution–precipitation mechanisms.

For the SrTiO_3_, the *in situ* transformation mechanism (Figure 1a) predicts that Sr^2+^ ions react with the surface of TiO_2_ nanoparticles, forming a SrTiO_3_ layer. As the Sr^2+^ ions diffuse and react with TiO_2_, the process continues until TiO_2_ is fully consumed. This mechanism can lead to the formation of core@shell or mesocrystal structures. The rate of this process is controlled by either the diffusion of Sr^2+^ ions through the SrTiO_3_ layer or the reaction between Sr^2+^ ions and TiO_2_.

**Figure 1:**
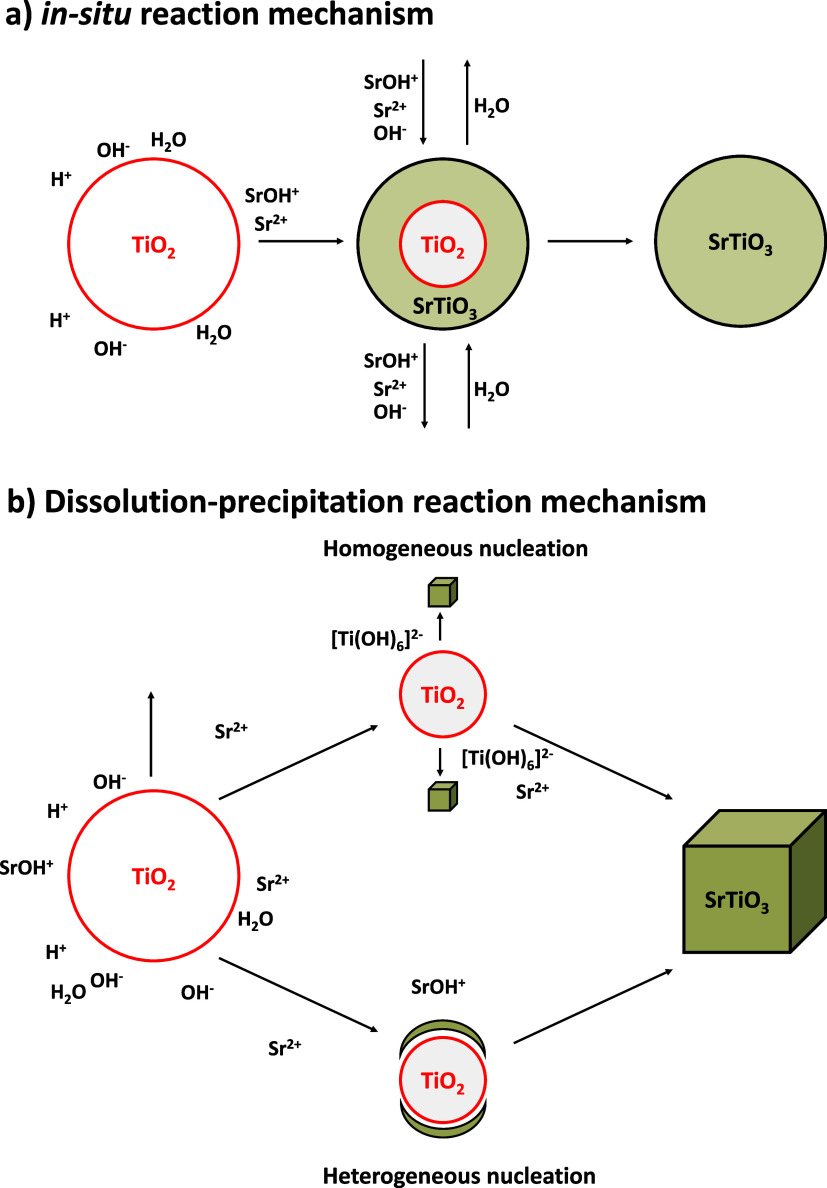
(a) *in situ* reaction mechanism and (b) dissolution–precipitation reaction mechanism for SrTiO_3_ formation.

Conversely, the dissolution–precipitation mechanism (Figure 1b) involves the dissolution of TiO_2_ and subsequent precipitation of SrTiO_3_.^23,24^ Homogeneous nucleation occurs when Ti–O bonds in TiO_2_ are hydrolyzed to form [Ti­(OH)_6_]^2–^, which then reacts with Sr^2+^ ions in solution to form SrTiO_3_. In heterogeneous nucleation, hydrated TiO_2_ promotes topotactic growth, where SrTiO_3_ nuclei form directly on the TiO_2_ surface.

The dissolution–precipitation mechanism is summarized by the following reactions:

$${\mathrm{TiO}}_{2\left(s\right)}+2H_{2}O\to \mathrm{Ti}{\left(\mathrm{OH}\right)}_{4\left(\mathrm{aq}\right)}$$ 5

$$\mathrm{Ti}{\left(\mathrm{OH}\right)}_{4\left(\mathrm{aq}\right)}+2{\mathrm{OH}}^{-}\to {\left[\mathrm{Ti}{\left(\mathrm{OH}\right)}_{6}\right]}^{2-}$$ 6

$${\left[\mathrm{Ti}{\left(\mathrm{OH}\right)}_{6}\right]}^{2-}+{\mathrm{Sr}}^{2+}\to {\mathrm{SrTiO}}_{3}↓+3H_{2}O$$ 7

Therefore, the nucleation rate of SrTiO_3_ depends on reactions 5 and 6, representing the dissolution rate of TiO_2_, and reaction 7, representing the precipitation rate of SrTiO_3_. If the nucleation rate were determined solely by reaction 7, complete conversion of TiO_2_ to SrTiO_3_ would be expected once all Ti would be in the form of [Ti­(OH)_6_]^2–^.

A hypothesis that amorphous TiO_2_ dissolves more easily than crystalline phases is therefore supported by the dissolution–precipitation mechanism. It corroborates with theoretical and experimental findings that demonstrate the solubility of Ti­(OH)_4_ species derived from amorphous TiO_2_ is significantly higher than that of anatase or rutile phases across a wide temperature range.^25^

This hypothesis was also supported by the influence of an alkaline medium in the reaction (Figure S2b). Higher pH favors TiO_2_ dissolution, accelerating SrTiO_3_ formation. Additionally, HRTEM images (Figure S3) confirm the hypothesis that SrTiO_3_ forms via dissolution–precipitation through homogeneous nucleation, as no core@shell structures, mesocrystals, epitaxial growth, or SrTiO_3_ nanoparticles onto TiO_2_ are observed.

The results strongly suggest that am-TiO_*x*_ is key to the production of SrTiO_3_. Therefore, we attempted to make the synthesis more cost-effective by studying the synthesis temperature within the range of 20 to 200 °C. As shown in Figure 2, the XRD patterns of SrTiO_3_ samples synthesized at temperatures between 20 and 200 °C with am-TiO_*x*_ as the precursor show diffraction peaks corresponding to the cubic SrTiO_3_ structure (JCPDS 35–0734). As the synthesis temperature increases, the intensity of the SrTiO_3_ diffraction peaks at 2θ ∼ 32° rises, accompanied by a noticeable shift toward higher diffraction angles (Figure 2b). Peaks corresponding to the SrCO_3_ structure (JCPDS 05–0418) are also observed, as it can be formed from the reaction of Sr^2+^ and CO_3_^2–^ that originates from dissolved CO_2_. SrCO_3_ precipitates even at low concentrations due to its low solubility (K_SP_ = 9.3.10^–10^).^26^ The enhanced peak intensity observed in Figure 2b is attributed to an improvement in crystallinity, while the shift to higher angles indicates a reduction in the lattice parameter (a_0_). Furthermore, nearly complete conversion of am-TiO_*x*_ to SrTiO_3_ is achieved at temperatures above 60 °C, highlighting the feasibility of using this method for SrTiO_3_ synthesis. Figure 2c presents the normalized intensity of I_(110)_, along with the unit cell volume and D_hkl_ values. The increase in temperature is followed by a decrease in volume and an increase in crystallinity, leading to the formation of crystals with shorter bond lengths. At 200 °C, the unit cell volume (59.67 Å^3^) approaches the ideal cubic value (59.54 Å^3^ from JCPDS 35–0734). Using the Scherrer eq (Table S2), we observed an overall increase in average crystallite size in the (110) plane, ranging from ∼29 to 42 nm.

**Figure 2:**
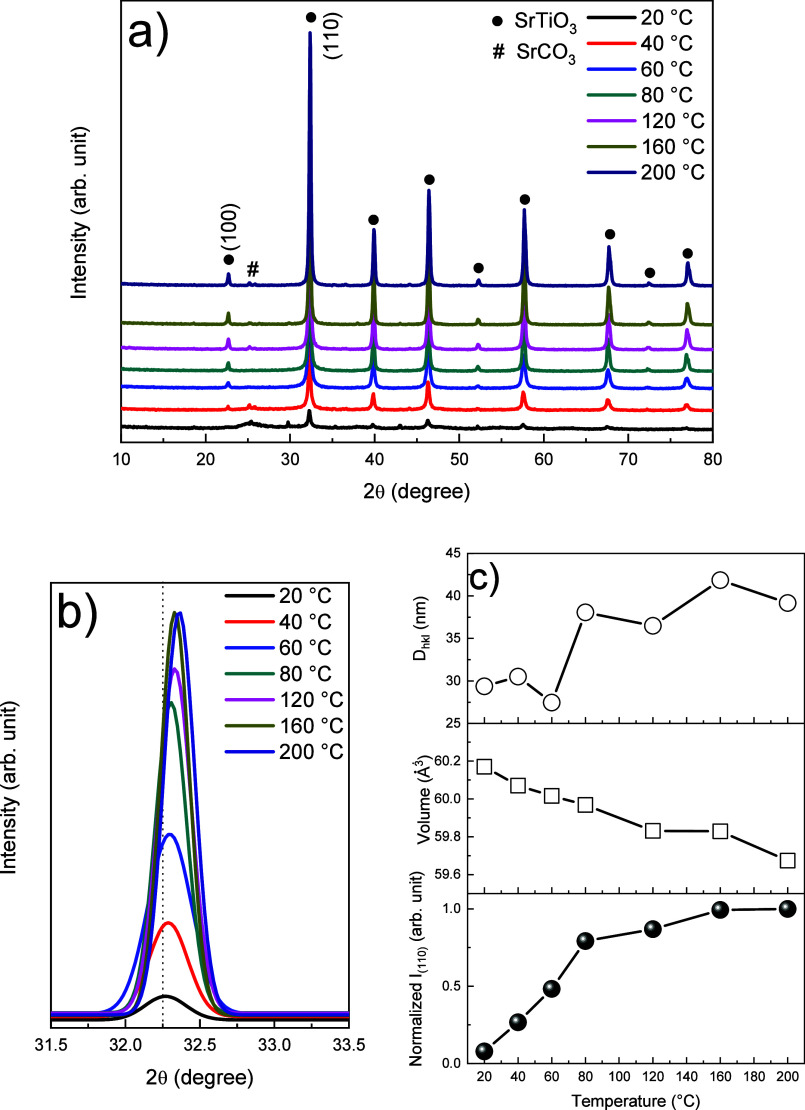
(a) XRD patterns of SrTiO_3_ samples obtained from 20 to 200 °C. (b) Detail of the peak at ∼32.2°. (c) Cell parameters and normalized intensity for I_(110)_.

The morphologies of the SrTiO_3_ samples obtained from am-TiO_*x*_ were evaluated using SEM and TEM (Figure 3). One can notice a clear evolution from nanocube-like morphology to rounded shapes when the temperature increases. At 20 °C (Figure 3a), the particles predominantly exhibit nanocube-like morphology with diameters of 158.2 ± 75.5 nm, with a broader size distribution (Figure S4). When the temperature reaches 80 °C, the morphology shifts to predominantly rounded shapes presenting a narrow size distribution and particles with an average diameter of 32.2 ± 15.7 nm. These nanostructures tend to aggregate due to their low colloidal stability, driven primarily by van der Waals forces.^27^

**Figure 3:**
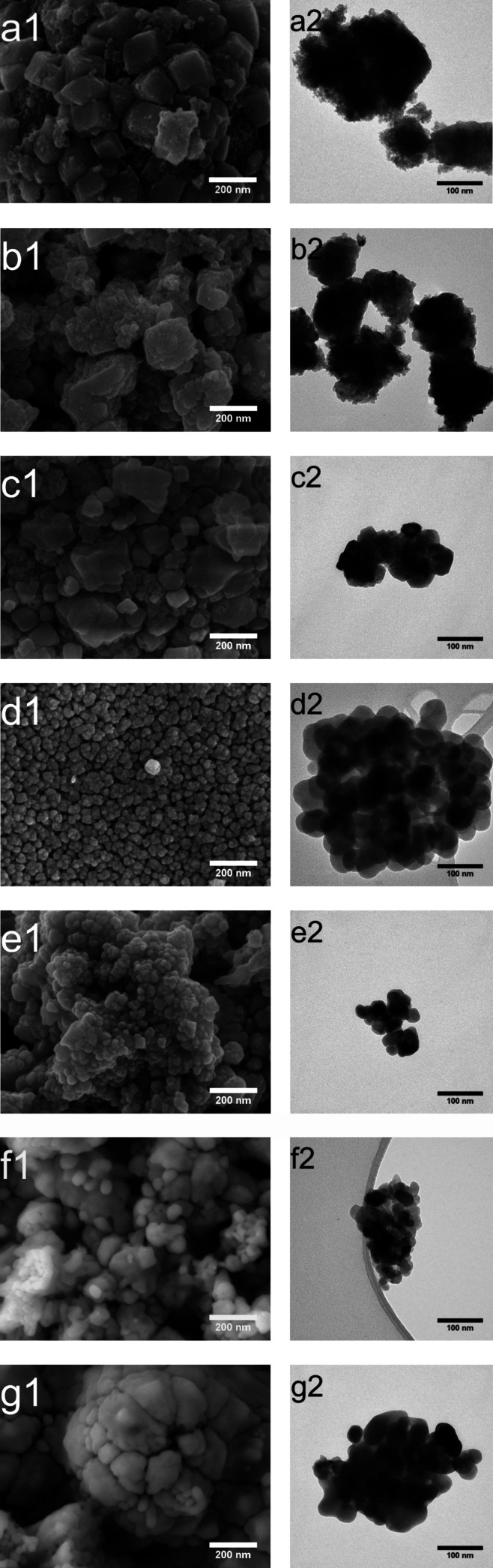
SEM (1) and TEM (2) images from SrTiO_3_ nanoparticles synthesized at (a) 20, (b) 40, (c) 60, (d) 80, (e) 120, (f) 160, and (g) 200 °C.

Figure 4 presents an HRTEM image of SrTiO_3_ synthesized at 200 °C using am-TiO_*x*_. The image in Figure 4 shows a highly crystalline nanoparticle with clear lattice fringes corresponding to the (110) plane, with an interplanar distance of 0.28 nm. The selected area electron diffraction (SAED) pattern in the inset of Figure 4 confirms the crystalline cubic perovskite structure of SrTiO_3_, as indicated by the distinct diffraction spots. These results corroborate the structural findings from XRD.

**Figure 4:**
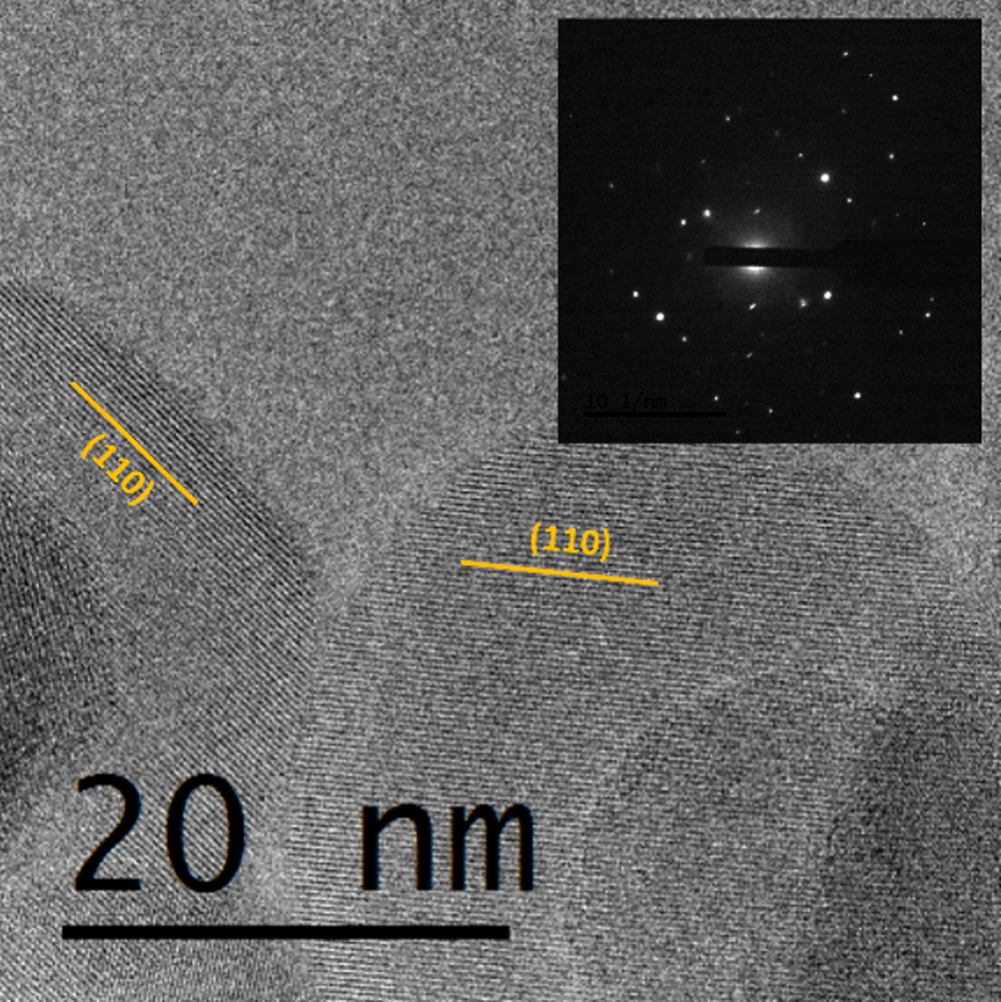
(a) HRTEM image of SrTiO_3_ nanoparticle obtained at 200 °C. Inset: SAED pattern.

At lower temperatures, the nanostructures become less organized, resulting in lower yields and less uniform morphology. Initial isotropic growth could lead to spherical particles, but anisotropic growth can occur due to differences in chemical bonding and atomic density between crystal facets. The reaction kinetics also support the proposed mechanism. The SrTiO_3_ synthesis at 200 °C under varying reaction times using am-TiO_*x*_ (Figure S5) demonstrates that perovskite formation occurs rapidly. A clear increase in crystallinity with corresponding changes in the cell parameters is observed within 15 min to 12 h.

The findings revealed by the XRD analysis suggest an evolution of cell parameters with increasing synthesis temperature. Therefore, it becomes important to investigate the local distortions in the crystal structure and the possible presence of defects using Raman spectroscopy.

Figure 5 presents the Raman spectra of the SrTiO_3_ samples. In its ideal cubic structure, SrTiO_3_ possesses a center of symmetry, and the polarizability of the crystal remains unchanged during atomic vibrations, resulting in the absence of Raman-active modes. However, as shown in Figure 5 and consistently reported in the literature, transverse optical (TO) and longitudinal optical (LO) modes of SrTiO_3_ are detected.^17,27–29^ This indicates a loss of symmetry due to structural imperfections, which in this case are attributed to synthesis temperature and may be associated with intrinsic defects or oxygen nonstoichiometry. The Raman spectra exhibit bands at ∼180, 288, 544, 727, and 793 cm^–1^, corresponding to the TO_2_, TO_3_, TO_4_, TO, and LO_4_ modes, respectively. The TO_2_ mode is linked to vibrations of Sr^2+^ cations relative to the TiO_6_ octahedra, while the TO_4_ mode involves bending vibrations of oxygen atoms within these octahedra. For synthesis temperatures below 40 °C, additional features are observed in the Raman spectra, which are not only assigned to the TO and LO modes of SrTiO_3_ but also to residual am-TiO_*x*_ precursor (as evidenced in Figure S1b), and to SrCO_3_, whose presence is more pronounced at lower synthesis temperatures.^30^ Furthermore, the broadening and position of Raman bands compared to the 200 °C sample suggests increased atomic site disorder, which disrupts translational and inversion symmetry.^31^ These local distortions reinforce the presence of crystal imperfections and confirm the structural disorder induced by the synthesis temperature.

**Figure 5:**
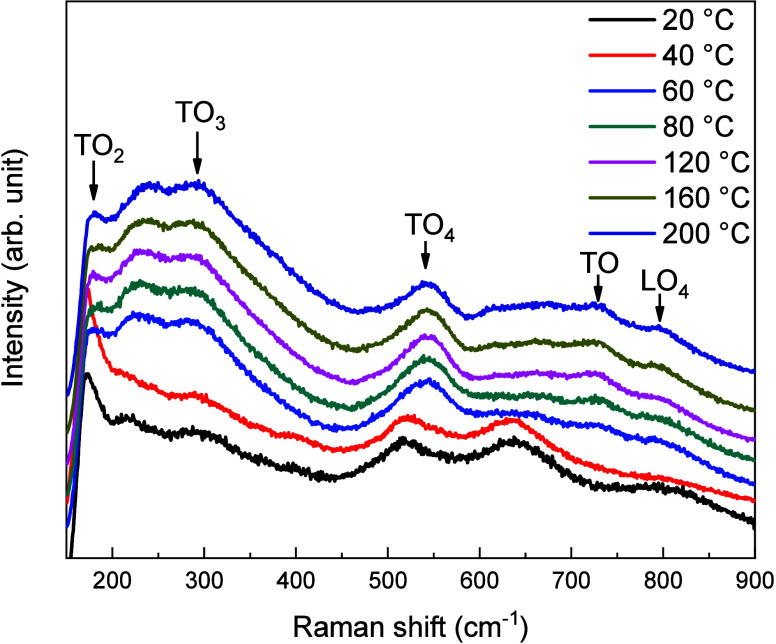
Raman spectroscopy of SrTiO_3_ nanoparticles obtained in the range of temperature from 20 to 200 °C.

The E_gap_ values of SrTiO_3_ synthesized at various temperatures were estimated from the diffuse reflectance spectra (Figure S6a), with the corresponding Kubelka–Munk plots shown in Figure S6b. The hydrothermal synthesis temperature had minimal effect on E_gap_, yielding values from 3.36 to 3.28 eV. This suggests that the presence of SrCO_3_ (especially at lower synthesis temperatures) and the differences in crystallinity among the samples do not significantly influence the optical E_gap_. These values are consistent with those reported for similar materials.^17,21,27,32^

XPS analyses were performed on samples synthesized at 60 and 200 °C to provide insights into the chemical bonds and valence states of surface atoms in SrTiO_3_ nanoparticles. Figure 6a presents the survey spectrum, with identifiable peaks corresponding to Sr, Ti, O, and C. The Ti 2p core-level XPS spectrum (Figure 6b) reveals Ti 2p_3/2_ (458.3 eV) and Ti 2p_1/2_ (464.0 eV) peaks, characteristic of Ti^4+^ species in stoichiometric SrTiO_3_.^33,34^ Additionally, a minor component at lower binding energy is associated with Ti^3+^, indicating the presence of structural defects such as oxygen vacancies.^21,33^ Notably, a lower hydrothermal treatment temperature leads to a higher content of Ti^3+^ species and an increase in the Ti^3+^/Ti^4+^ ratio.

**Figure 6:**
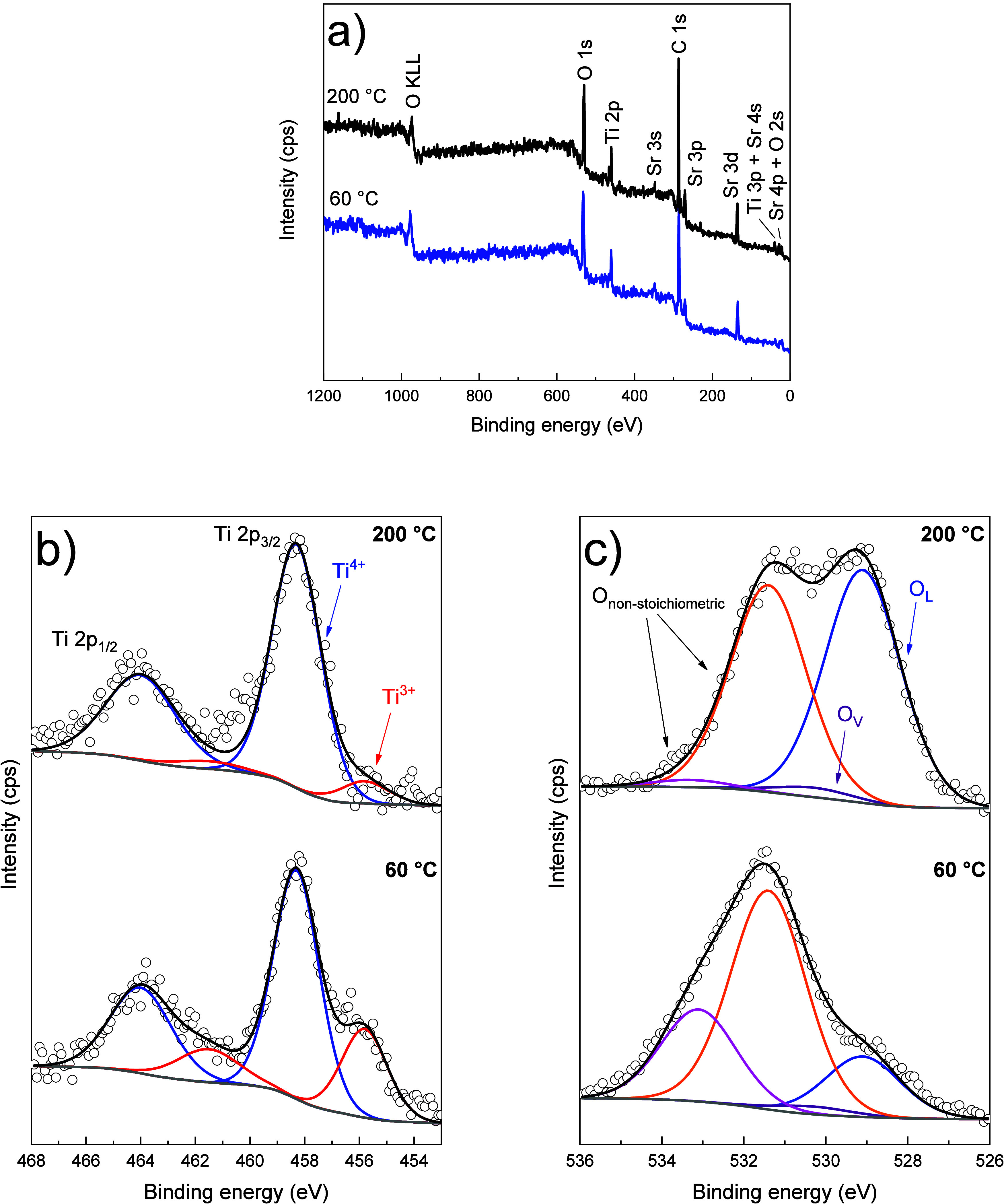
XPS spectrum of SrTiO_3_ obtained at 200 °C. (a) Long scan, (b) Ti 2p and (c) O 1s core-level XPS.

Further evidence of an increased oxygen vacancy concentration in the sample synthesized at a shorter reaction time is provided by the O 1s spectra (Figure 6c). The O 1s core-level spectrum displays four components: lattice oxygen (O_L_) at 529.1 eV, oxygen vacancies (O_V_) at 530.3 eV, and other two components at 531.4 and 533.3 eV that can be attributed to nonstoichiometric/adsorbed species (including both organic and hydroxyl groups), carbonates and metal-hydroxides.^21,33,35,36^ While peak fitting can lead to varying interpretations, it is qualitatively evident that the sample synthesized at 200 °C exhibits a higher (O_L_+O_V_)/(nonstoichiometric species) ratio. This observation aligns with the Ti 2p spectra findings, as a higher Ti^3+^ content corresponds to an increased presence of adsorbed oxygen species. Table S3 summarizes the results from the fitting of XPS regions. The Sr 3d spectrum and C 1s spectrum are presented in Figure S7 and support these findings.^4^

We finally investigated the photocatalytic activity of the SrTiO_3_-based samples for H_2_ production under solar light irradiation. Figure 7a compares the amount of evolving H_2_ gas for all samples, while Figure 7b summarizes the corresponding H_2_ evolution rates. The photocatalytic activity of bare SrTiO_3_ (without cocatalyst) is provided in Figure S8. The highest H_2_ evolution rate was observed in the sample synthesized at 60 °C, reaching approximately 43 μmol h^–1^. This value represents an improvement of approximately 8.6 times compared to the sample synthesized at 200 °C, which exhibits the highest crystallinity and the narrowest size distribution among the samples. Table S4 summarizes the experimental conditions, crystallographic parameters, and H_2_ evolution rates obtained in this study, while Table S5 presents a comparative overview of H_2_ production rates reported in the literature for similar inorganic semiconductors.

**Figure 7:**
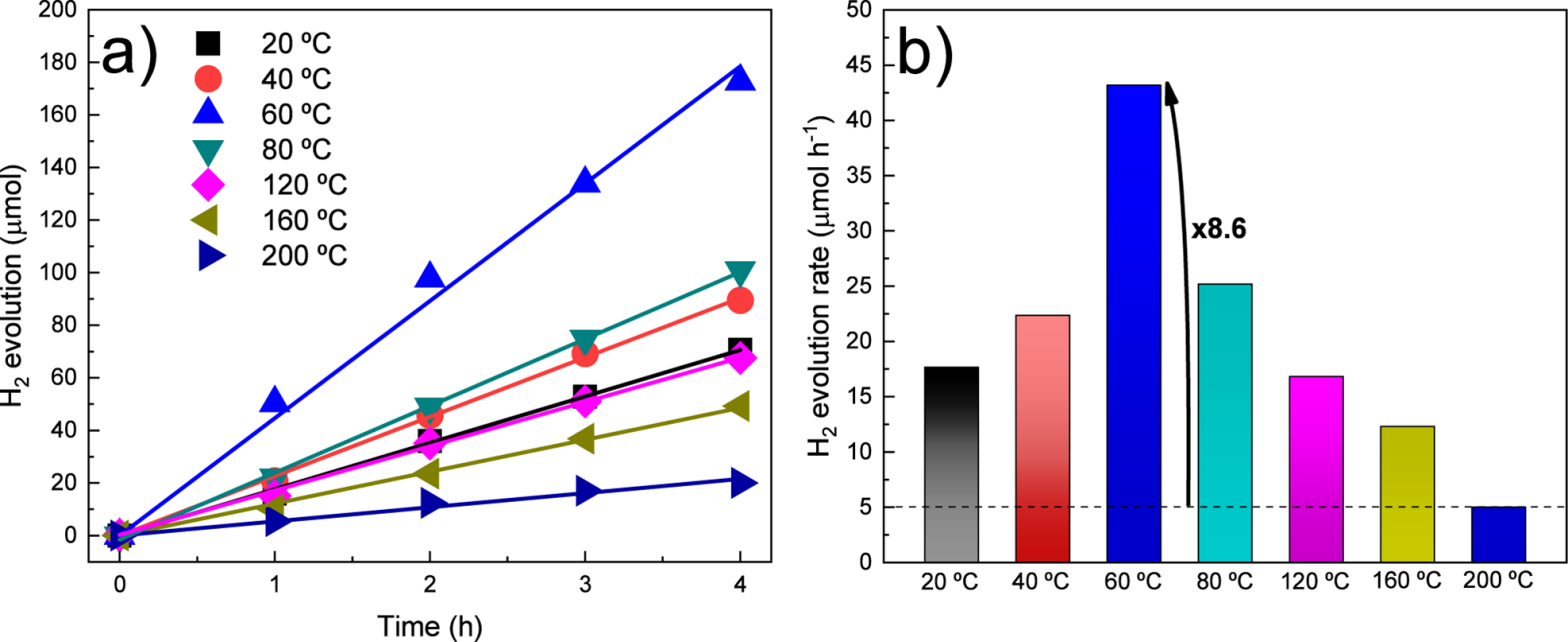
Photocatalytic H_2_ (a) evolution and (b) evolution rate for SrTiO_3_-based samples.

Therefore, the controlled hydrothermal synthesis temperature not only improved the crystallinity of the material but also contributed to a reduction in average particle size. Since photocatalytic efficiency is influenced by multiple factors,^21,37–39^ including surface area, crystallinity, and composition, our results indicated that the enhanced performance of the SrTiO_3_-based samples arises from the synergistic interplay among these properties. This combination leads to the highest hydrogen evolution activity for the sample synthesized at a relatively low temperature of 60 °C.

All SrTiO_3_-based samples were modified with Ru-based cocatalyst prior to the photocatalytic tests. The presence of a cocatalyst plays a crucial role in enhancing the photocatalytic performance by acting as an efficient electron sink, promoting charge separation and facilitating the hydrogen evolution reaction.^40^ This modification also minimizes surface recombination effects and improves interfacial charge transfer, enabling a more accurate comparison of the intrinsic photocatalytic activity of the SrTiO_3_ samples synthesized at different temperatures. Additionally, the presence of SrCO_3_ may further enhance interfacial charge dynamics by facilitating charge carrier transfer from SrTiO_3_ to SrCO_3_, as reported before.^4,35^ This may help explain the appreciable H_2_ production observed in the low-temperature samples, despite their comparatively lower crystallinity.

The proposed formation mechanism and its photocatalytic behavior are schematically represented in Figure 8. Nucleation is driven by the dissolution rate and is strongly influenced by the crystalline phase of the Ti precursor. The scheme in Figure 8 illustrates the dissolution of am-TiO_*x*_ (A-TiO_2_ or R-TiO_2_) into [Ti­(OH)_6_]^2–^, followed by its reaction with Sr^2+^, facilitating the nucleation and growth of SrTiO_3_ nanoparticles. The interplay of crystallinity, morphology, and composition determines the photocatalytic performance, with the sample synthesized at 60 °C exhibiting the highest water reduction efficiency among all samples, further demonstrating the viability of this approach.

**Figure 8:**
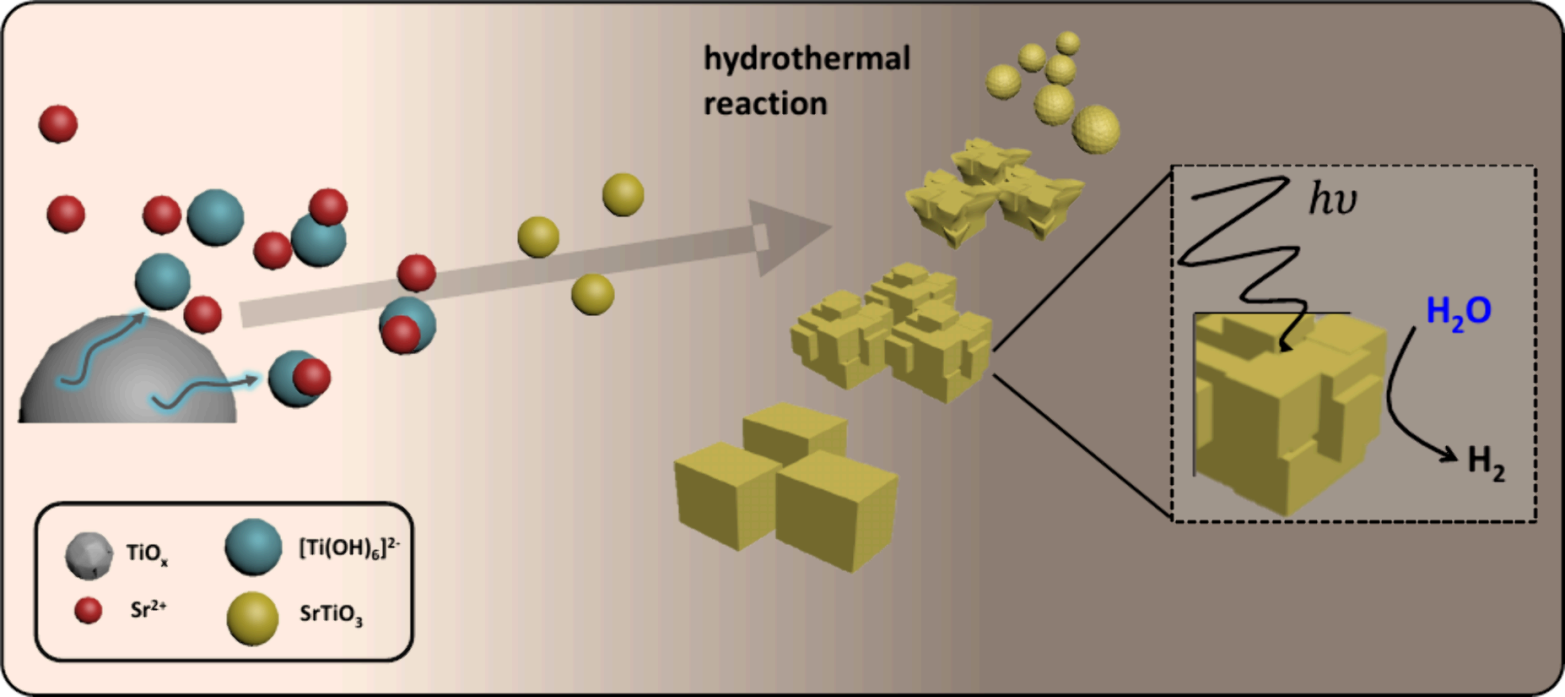
Schematic illustration of the proposed dissolution–precipitation mechanism through homogeneous nucleation of SrTiO_3_ nanoparticles and its photocatalytic application for H_2_ production.

## Conclusions

In summary, our investigation into the hydrothermal synthesis of SrTiO_3_ nanoparticles using polymorphic titanium precursors has highlighted the critical role of synthesis temperature in modulating the structural, morphological, and chemical features of the final material. The findings reveal a clear trend of increased crystallinity and reduced defect density with increasing temperature, accompanied by notable morphology evolution. The proposed dual-pathway formation mechanism underscores the significance of precursor solubility and reactivity, demonstrating the advantages of amorphous titanium precursors in achieving improved control over particle morphology and crystallinity. This study contributes to a deeper understanding of metal oxide synthesis and provides a valuable framework for optimizing material properties for photocatalysis and related applications.

## Supplementary Material



## Notes

Experimental details; Tables; physiochemical characterization of the samples: structural characterization from XRD; size distribution of the particles; SEM and TEM images; UV–vis absorption spectra, and hydrogen production (PDF)
