# Supplementary material for: Engineering SrTiO3 Nanostructures for Enhanced Photocatalytic Performance: Unveiling the Influence of Titanium Precursors and Synthesis Temperature
Source: ACS Omega. 2025 Aug 28;10(35):40066–75. doi: 10.1021/acsomega.5c04899 (PMC12423891; doi:10.1021/acsomega.5c04899)
Supplement: Supplementary file 1 [file ao5c04899_si_001.pdf]

**Supporting information**

# Engineering $SrTiO_3$ Nanostructures for Enhanced Photocatalytic Performance: Unveiling the Influence of Titanium Precursors and Synthesis Temperature

Anderson Thesing,[a*] Lara F. Loguercio,[b] Edjan Alves da Silva,[a] Gabriel Franciosi,[a] Arturo B. L. Véliz,[b] Muhammad R. K. Khattak,[a] Alexandre G. Brolo,[c] Marcos J. L. Santos,[b] and Jacqueline F. L. Santos[b*]

[a]Institute of Physics, Universidade Federal do Rio Grande do Sul, Av. Bento Gonçalves 9500, Bairro Agronomia, Porto Alegre – RS, 91501-970, Brazil

[b]Institute of Chemistry, Universidade Federal do Rio Grande do Sul, Av. Bento Gonçalves 9500, Bairro Agronomia, Porto Alegre – RS, 91501-970, Brazil

[c]Department of Chemistry and Center for Advanced Materials and Related Technologies, University of Victoria, P.O. Box 3065, Victoria – British Columbia, V8W 3V6, Canada

*Corresponding authors: anderson.thesing@ufrgs.br; jacqueline.ferreira@ufrgs.br

From the XRD in Figure S1a, a broad halo is observed for am-$TiO_x$ with no defined peaks, indicating the amorphous nature of the sample. At 420 °C, only the anatase phase is present (indicated as A, according to JCPDS 21-1272), while at 700 °C only rutile phase is present (indicated as R, according to JCPDS 76-1940).

Raman spectroscopy results are presented in Figure S1b. The bands at 193, 392, 511, and 636 $cm^{-1}$ (corresponding to the $E_g$, $B_{1g}$, $A_{1g}$+$B_{1g}$, and $E_g$ modes, respectively) are attributed to the anatase phase, while the bands at 443 and 605 $cm^{-1}$ (corresponding to the $E_g$ and $A_{1g}$ modes, respectively) are assigned to the rutile phase. The band at 230 $cm^{-1}$ is attributed to second-order modes.[1–3] The am-$TiO_2$ sample exhibits broad, poorly defined bands, characteristic of an amorphous structure.

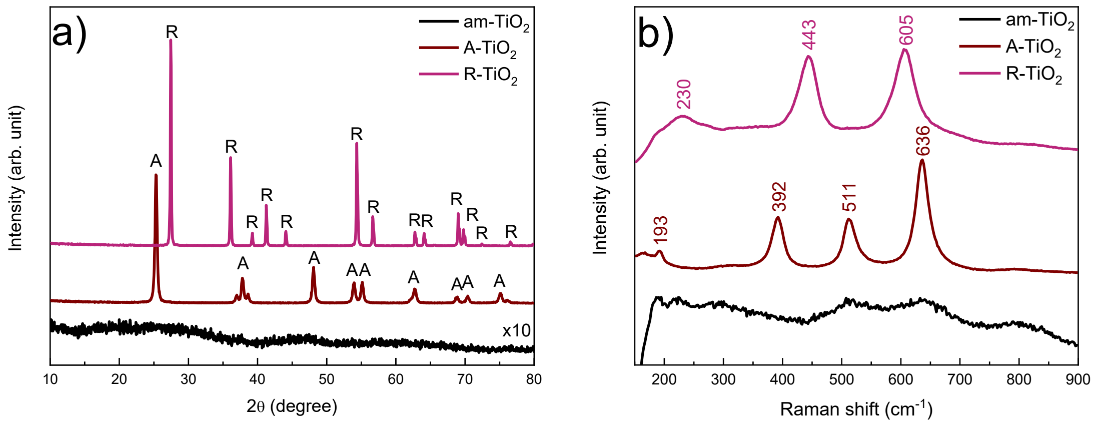


Figure S1. (a) XRD and (b) Raman of $TiO_2$ polymorphs.

**Table S1.** Experimental conditions used for the synthesis of $SrTiO_3$ from polymorphic $TiO_2$.

| **Polymorphic $TiO_2$** | **Temperature of hydrothermal reaction (°C)** | **NaOH** (mol $L^{-1}$) | **Reaction time (min)** |
|---|---|---|---|
| Amorphous | 20 | - | 720 |
| Amorphous | 40 | - | 720 |
| Amorphous | 60 | - | 720 |
| Amorphous | 80 | - | 720 |
| Amorphous | 120 | - | 720 |
| Amorphous | 160 | - | 720 |
| Amorphous | 200 | - | 720 |
| Anatase | 160 | - | 720 |
| Anatase | 160 | 0.01 | 720 |
| Rutile | 160 | - | 720 |
| Rutile | 160 | 0.01 | 720 |
| Amorphous | 200 | - | 15 |
| Amorphous | 200 | - | 30 |
| Amorphous | 200 | - | 60 |
| Amorphous | 200 | - | 120 |

The dissolution of $TiO_2$ can be modulated by adjusting the pH of the reaction medium, with higher pH values promoting increased $TiO_2$ solubility. XRD analysis of hydrothermal reactions conducted in 0.01 mol $L^{-1}$ NaOH solution, using anatase $TiO_2$ (A-$TiO_2$) and rutile $TiO_2$ (R-$TiO_2$) as precursors, reveals differing conversion efficiencies (Figure S2b). In the absence of NaOH, $SrTiO_3$ formation is more favorable when A-$TiO_2$ is used. The addition of NaOH enhances $SrTiO_3$ formation for both precursors, with a more pronounced increase observed for A-$TiO_2$. As previously discussed by Hanaor and Sorrell,[4] $TiO_2$ in the rutile phase exhibits higher thermodynamic stability over a broad range of pressures and temperatures (0 to 2500 K), which likely accounts for the lower reactivity and reduced conversion efficiency of R-$TiO_2$ under the same conditions.

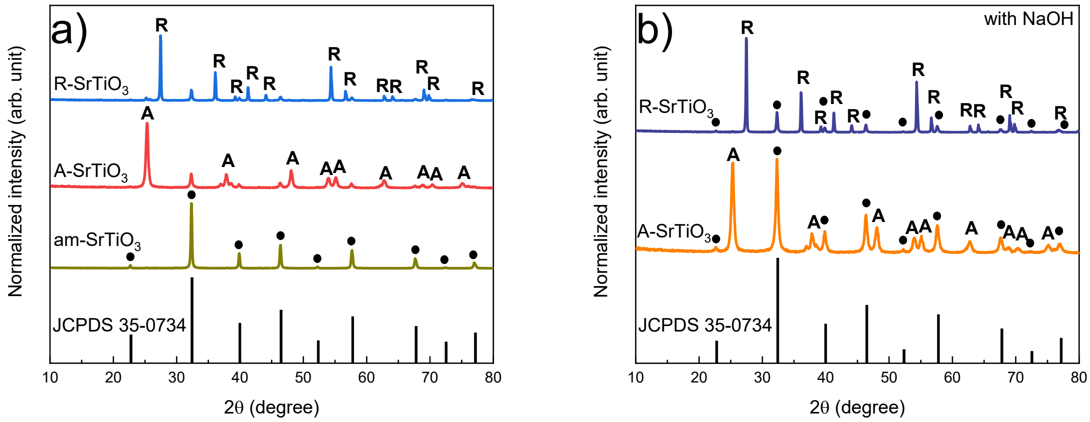


Figure S2. XRD patterns of $SrTiO_3$ synthesized at 160 °C for 12 hours. (a) without NaOH and (b) with NaOH using amorphous or crystalline $TiO_2$. Anatase phase is indicated as A according to JCPDS 21-1272, rutile phase is indicated as R according to JCPDS 76-1940, and $SrTiO_3$ (black bars) is indicated as ● according to JCPDS 35-0734.

HRTEM images of $SrTiO_3$ synthesized at 160 °C using A-$TiO_2$ as titanium source and NaOH show distinct nanoparticles with adjacent crystal planes at distances of 0.35 nm and 0.28 nm, corresponding to the (101) planes of A-$TiO_2$ and the (110) planes of $SrTiO_3$, respectively.

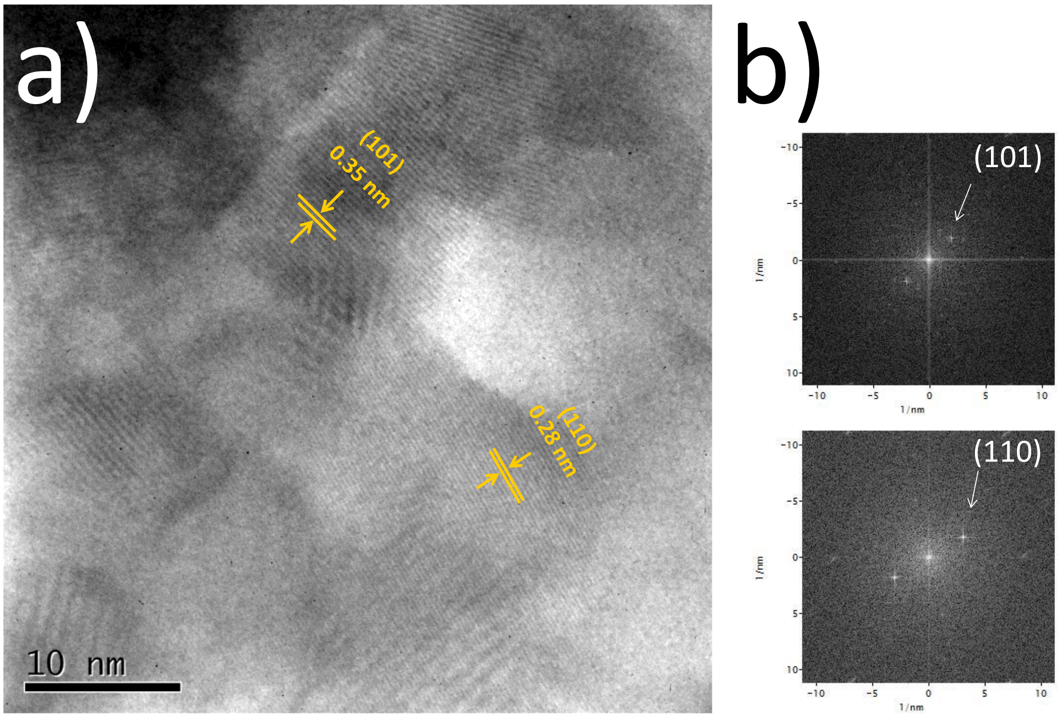


Figure S3. (a) HRTEM of $SrTiO_3$ synthesized at 160 °C for 12 hours with NaOH using A-$TiO_2$ as the titanium source. (b) FFT images.

**Table S2.** Data used for calculating $D_{hkl}$ from Scherrer equation along (110) plane. $K = 0.89$ and $\lambda = 1.54$ Å.

| **Temperature of hydrothermal reaction (°C)** | **Reaction time (min)** | $\boldsymbol{\beta}$ **(rad)** | $\boldsymbol{\theta}$ **(rad)** | $\boldsymbol{D_{hkl}}$ **(nm)** |
|---|---|---|---|---|
| 20 | 720 | 0.00486 | 0.28160 | 29.36 |
| 40 | 720 | 0.00468 | 0.28176 | 30.51 |
| 60 | 720 | 0.0052 | 0.28185 | 27.45 |
| 80 | 720 | 0.00375 | 0.28193 | 38.07 |
| 120 | 720 | 0.00391 | 0.28215 | 36.48 |
| 160 | 720 | 0.00341 | 0.28215 | 41.85 |
| 200 | 720 | 0.00364 | 0.28240 | 39.16 |
| 200 | 15 | 0.00557 | 0.28180 | 25.61 |
| 200 | 30 | 0.00494 | 0.28194 | 28.86 |
| 200 | 60 | 0.00417 | 0.28203 | 34.26 |
| 200 | 120 | 0.00412 | 0.28215 | 34.61 |

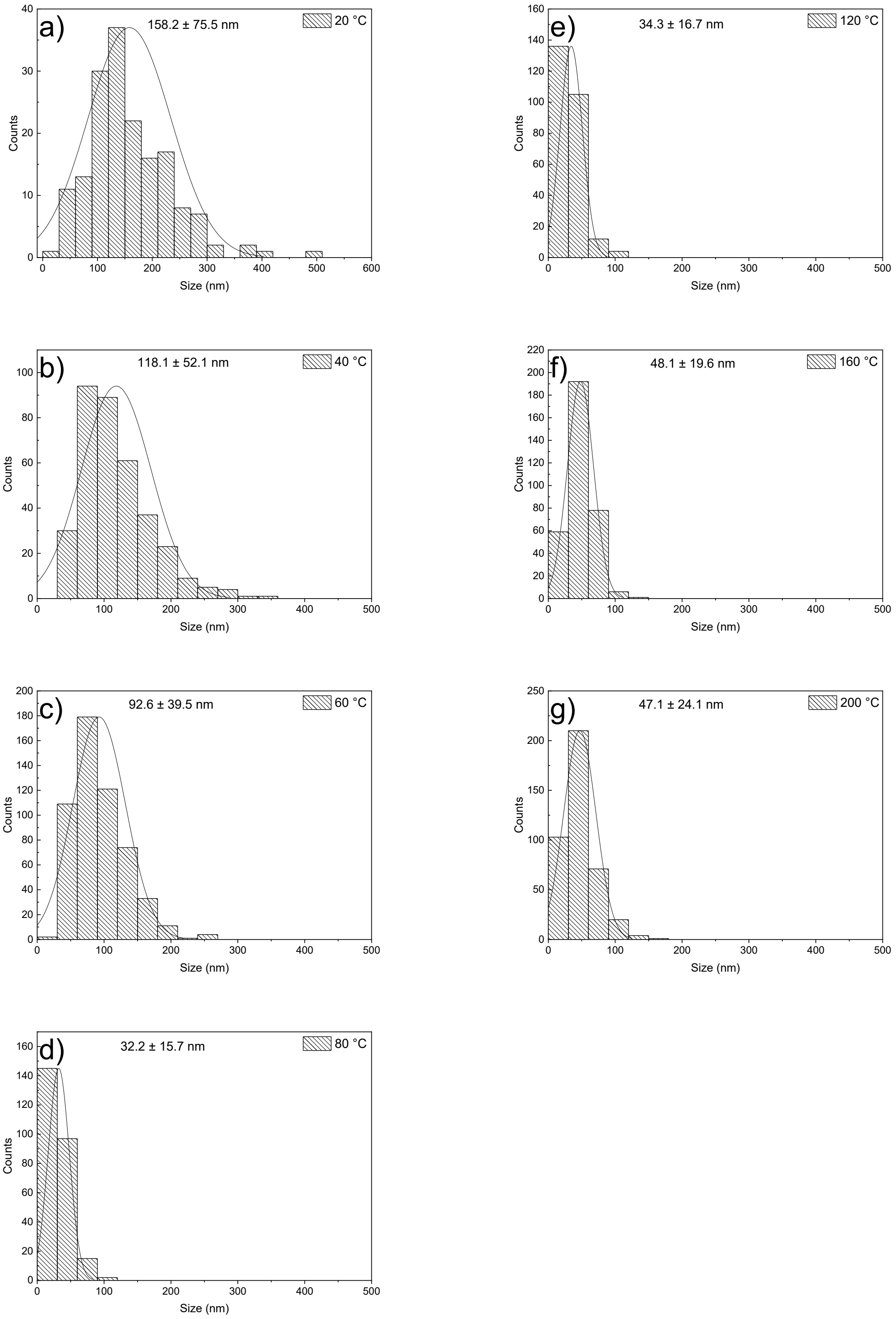


Figure S4. (a-f) Particles size distribution of $SrTiO_3$ samples obtained from 20 to 200 °C.

XRD analysis of $SrTiO_3$ synthesized at 200 °C at different times shows a shift in the (110) diffraction plane to higher values (Figure S5b), indicating a reduced $a_0$ and an increase in peak intensity, both of which are strongly time-dependent (Figure S5c).

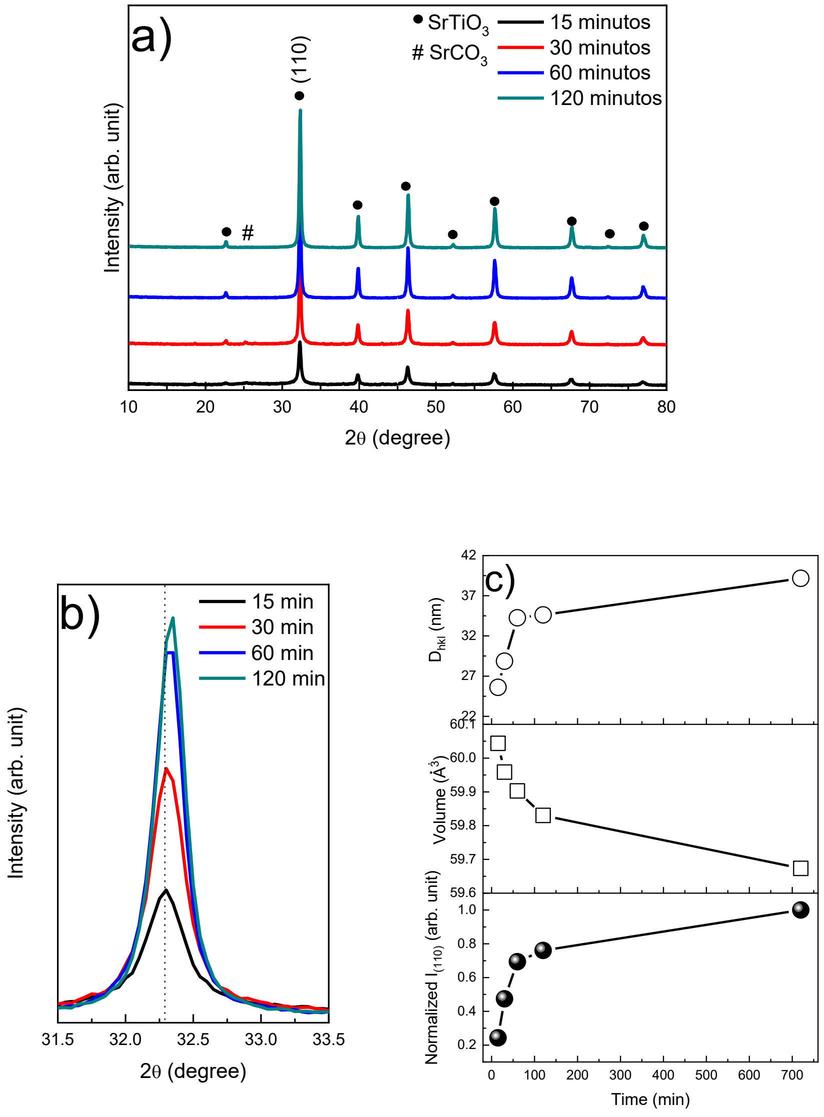


Figure S5. (a) XRD patterns of the $SrTiO_3$ synthesized at 200 °C with hydrothermal reaction times from 15 to 120 minutes. (b) Detail of the shift in the (110) peak. (c) Variation in the intensity of the (110) peak and the lattice parameter $a_0$ for the (110). For comparison, data from the sample synthesized after 12 hours (720 min) is included.

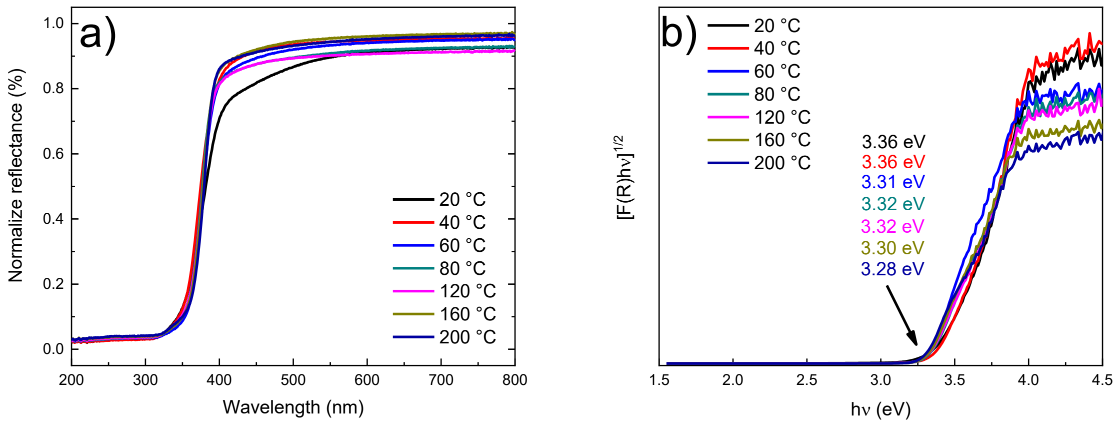


Figure S6. (a) Diffuse reflectance spectra and (b) Kubelka-Munk plot of $SrTiO_3$ nanoparticles obtained from 20 to 200 °C.

**Table S3.** Results from the fitting of XPS regions.

| Assigment | Chemical component | Sample | | | |
|---|---|---|---|---|---|
| | | 60 °C | | 200 °C | |
| | | BE (eV) | %area | BE (eV) | %area |
| Ti 2p | $Ti^{4+}$ (Ti $2p_{3/2}$) | 458.3 | 47.99 | 458.3 | 61.05 |
| | $Ti^{4+}$ (Ti $2p_{1/2}$) | 464.0 | 24.10 | 464.0 | 30.65 |
| | $Ti^{3+}$ (Ti $2p_{3/2}$) | 455.8 | 18.58 | 455.8 | 5.53 |
| | $Ti^{3+}$ (Ti $2p_{1/2}$) | 461.5 | 9.33 | 461.5 | 2.77 |
| O 1s | $O_L$ (lattice) | 529.1 | 15.9 | 529.1 | 50.59 |
| | $O_V$ (vacancies) | 530.3 | 2.07 | 530.3 | 2.09 |
| | $O_{non\text{-}stoichiometric}$ | 531.4 | 57.71 | 531.4 | 45.36 |
| | $O_{non\text{-}stoichiometric}$ | 533.1 | 24.33 | 533.3 | 1.59 |
| $Ti^{3+}/Ti^{4+}$ | | 0.387 | | 0.09 | |
| $(O_L+O_V)/(O_{non\text{-}stoichiometric\ species})$ | | 0.219 | | 1.122 | |

In the Sr 3d spectrum (Figure S7a), the Sr $3d_{5/2}$ and Sr $3d_{3/2}$ doublet corresponding to stoichiometric $Sr^{2+}$ species appear at a lower binding energy compared to any other possible Sr-based species. Notably, a higher-energy component becomes more pronounced at lower synthesis temperatures, indicating an increased presence of Sr-based species in the sample. The C 1s spectrum (Figure S7b) follows the same trend, confirming a minor contribution from $SrCO_3$ in the sample synthesized at a higher temperature.

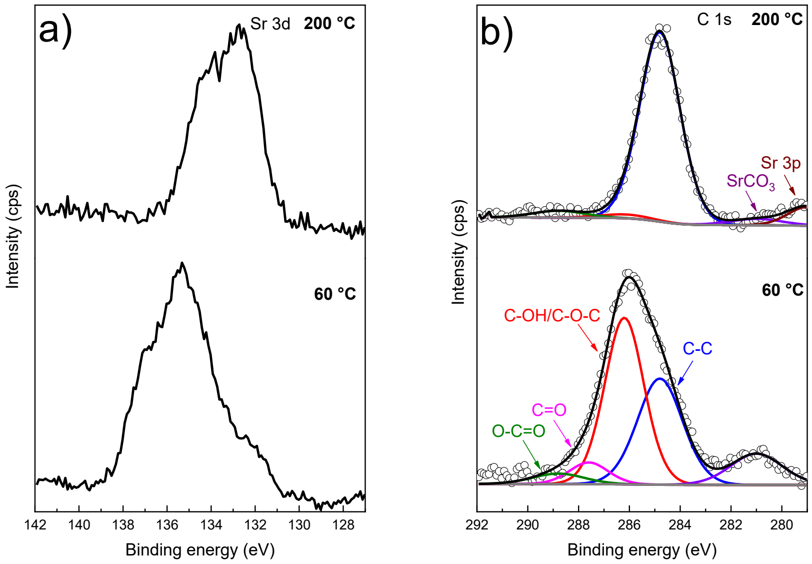


Figure S7. (a) Sr 3d and (b) C 1s core-level XPS.

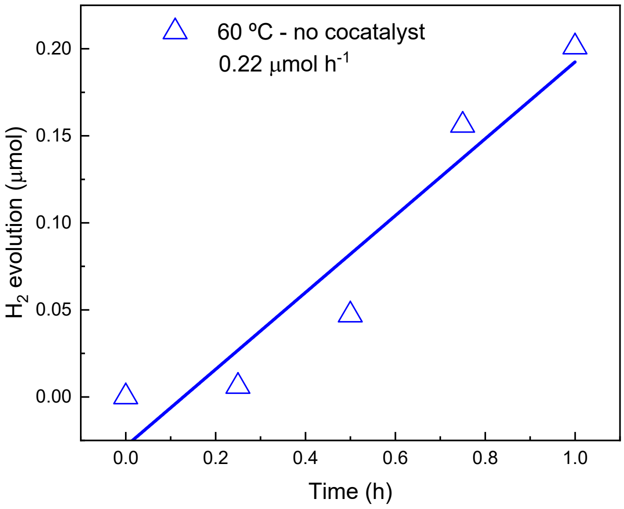


Figure S8. Photocatalytic $H_2$ evolution for bare $SrTiO_3$ (without cocatalyst) nanoparticles.

**Table S4.** Summary of experimental conditions, cell parameters and $H_2$ evolution rate.

| **Temperature of hydrothermal reaction (°C)** | $\boldsymbol{D_{hkl}}$ **(nm)** | $\boldsymbol{a_0}$ **(Å$^3$)** | **$E_{gap}$ (eV)** | **$H_2$ evolution rate (μmol h$^{-1}$)** |
|---|---|---|---|---|
| 20 | 29.36 | 60.17 | 3.36 | 17.66 |
| 40 | 30.51 | 60.07 | 3.36 | 22.37 |
| 60 | 27.45 | 60.02 | 3.31 | 43.19 |
| 80 | 38.07 | 59.97 | 3.32 | 25.20 |
| 120 | 36.48 | 59.83 | 3.32 | 16.82 |
| 160 | 41.85 | 59.83 | 3.30 | 12.31 |
| 200 | 39.16 | 59.67 | 3.28 | 5.00 |

**Table S5.** Comparison of photocatalysts on $H_2$ production rate.

| Photocatalyst | Reaction media | $H_2$ evolution rate ($\mu mol\ h^{-1}$) | Ref. |
|---|---|---|---|
| $CuS/SrTiO_3$ | 20% vol. methanol aqueous solution | 20.4 | 5 |
| $CuO/SrTiO_3$ | 60% vol. methanol aqueous solution | 192.5 | 6 |
| $Cu/TiO_2$ nanorods | 20% vol. methanol aqueous solution | 1023.8 | 7 |
| $Pt/SrTiO_3$ | 20% vol. triethanolamine aqueous solution | 316.1 | 8 |
| $Pt/SrTiO_3$ | methanol aqueous solution | 131.4 | 9 |
| $FeO_x$-$Pt/SrTiO_3$ | methanol aqueous solution | 254.5 | 9 |
| $SrTiO_3$ | 25% vol. ethanol aqueous solution | 8.78 | 10 |
| $SrTiO_{3-x}$ | 10% vol. triethanolamine aqueous solution | 8.25 | 11 |
| $Pt/SrTiO_{3-x}$ | 25% vol. methanol aqueous solution | 110 | 12 |
| $LiNbO_3$ | 25% vol. ethanol aqueous solution | 0.10 | 13 |
| $RuO_x/SrTiO_3$ | 10% vol. methanol aqueous solution | 43 | *This work |

## References


1. Wypych, A. *et al.* Dielectric Properties and Characterisation of Titanium Dioxide Obtained by Different Chemistry Methods. *J Nanomater* **2014**, (2014).

2. Zhang, Y., Harris, C. X., Wallenmeyer, P., Murowchick, J. & Chen, X. Asymmetric Lattice Vibrational Characteristics of Rutile $TiO_2$ as Revealed by Laser Power Dependent Raman Spectroscopy. *The Journal of Physical Chemistry C* **117**, 24015–24022 (2013).

3. W F Zhang, Y L He, M S Zhang, Z Yin & Q Chen. Raman scattering study on anatase $TiO_2$ nanocrystals. *J Phys D Appl Phys* **33**, 912–916 (2000).

4. Hanaor, D. A. H. & Sorrell, C. C. Review of the anatase to rutile phase transformation. *J Mater Sci* **46**, 855–874 (2011).

5. Zhou, D. *et al.* CuS co-catalyst modified hydrogenated $SrTiO_3$ nanoparticles as an efficient photocatalyst for $H_2$ evolution. *Dalton Transactions* **50**, 7768–7775 (2021).

6. Mai, X. T. *et al.* Effect of CuO Loading on the Photocatalytic Activity of SrTiO3 for Hydrogen Evolution. *Inorganics (Basel)* **10**, 130 (2022).

7. Chen, W. *et al.* Non-noble metal Cu as a cocatalyst on TiO 2 nanorod for highly efficient photocatalytic hydrogen production. *Appl Surf Sci* **445**, 527–534 (2018).

8. Souri, A. P., Skliri, E., Vamvasakis, I., Armatas, G. S. & Binas, V. Highly active pt nanoparticles supported on SrTiO3 for photocatalytic hydrogen production. *Applied Physics A* **130**, 785 (2024).

9. Zhai, M. *et al.* Modification of Pt co-catalyst on SrTiO3 with amorphous FeOx towards enhanced photocatalytic overall water splitting. *J Catal* **443**, 115989 (2025).

10. Thesing, A. *et al.* Surface Oxygen Vacancies in Hierarchical Metal Oxide Nanostructures: Optoelectronic Tuning in Photocatalysis. *The Journal of Physical Chemistry C* **128**, 21479–21487 (2024).

11. Li, C. Q. *et al.* Oxygen vacancy engineered SrTiO3 nanofibers for enhanced photocatalytic H2 production. *J Mater Chem A Mater* (2019) doi:10.1039/c9ta03701b.

12. Tan, H. *et al.* Oxygen vacancy enhanced photocatalytic activity of pervoskite SrTiO3. *ACS Appl Mater Interfaces* (2014) doi:10.1021/am5051907.

13. Deon, V. G. *et al.* Synthesis of LiNbO3 nanocrystals by microwave-assisted hydrothermal method: formation mechanism and application to hydrogen evolution reaction. *Chemical Papers* **75**, 3807–3815 (2021).
